# Complete genome sequence of *Corynebacterium pseudotuberculosis* biovar ovis strain P54B96 isolated from antelope in South Africa obtained by rapid next generation sequencing technology

**DOI:** 10.4056/sigs.3066455

**Published:** 2012-12-15

**Authors:** Syed Shah Hassan, Luis Carlos Guimarães, Ulisses de Pádua Pereira, Arshad Islam, Amjad Ali, Syeda Marriam Bakhtiar, Dayana Ribeiro, Anderson Rodrigues dos Santos, Siomar de Castro Soares, Fernanda Dorella, Anne Cybelle Pinto, Maria Paula Cruz Schneider, Maria Silvanira Barbosa, Síntia Almeida, Vinícius Abreu, Flávia Aburjaile, Adriana Ribeiro Carneiro, Louise Teixeira Cerdeira, Karina Fiaux, Eudes Barbosa, Carlos Diniz, Flavia S. Rocha, Rommel Thiago Jucá Ramos, Neha Jain, Sandeep Tiwari, Debmalya Barh, Anderson Miyoshi, Borna Müller, Artur Silva, Vasco Azevedo

**Affiliations:** 1 Laboratório de Genética Celular e Molecular, Departamento de Biologia Geral, Instituto de Ciências Biológicas (ICB), Universidade Federal de Minas Gerais, Belo Horizonte, Brazil.; 2 Instituto de Ciências Biológicas, Universidade Federal do Pará, Belém, PA, Brazil.; 3 DST/NRF Centre of Excellence for Biomedical Tuberculosis Research/MRC Centre for Molecular and Cellular Biology, Division of Molecular Biology and Human Genetics, Faculty of Health Sciences, Stellenbosch University, Cape Town, South Africa.; 4 Centre for Genomics and Applied Gene Technology, Institute of Integrative Omics and Applied Biotechnology (IIOAB), Nonakuri, Purba Medinipur, West Bengal, India.; 5 Departamento de Medicina Veterinária, Universidade Federal de Lavras, Lavras, Brazil.; 6 Instituto de Ciências Exatas (ICEX), Universidade Federal de Minas Gerais, Belo Horizonte, MG, Brazil.

**Keywords:** **s**: biovar *ovis*, Gram-positive pathogen, caseous lymphadenitis/cheesy gland disease, liver lesion, Antelope, genome sequencing, Ion Torrent

## Abstract

The *Actinobacteria*, *Corynebacterium pseudotuberculosis* strain P54B96, a nonmotile, non-sporulating and a mesophile bacterium, was isolated from liver, lung and mediastinal lymph node lesions in an antelope from South Africa. This strain is interesting in the sense that it has been found together with non-tuberculous mycobacteria (NTMs) which could nevertheless play a role in the lesion formation. In this work, we describe a set of features of *C. pseudotuberculosis* P54B96, together with the details of the complete genome sequence and annotation. The genome comprises of 2.34 Mbp long, single circular genome with 2,084 protein-coding genes, 12 rRNA, 49 tRNA and 62 pseudogenes and a G+C content of 52.19%. The analysis of the genome sequence provides means to better understanding the molecular and genetic basis of virulence of this bacterium, enabling a detailed investigation of its pathogenesis.

## Introduction

Caseous lymphadenitis (CLA) or cheesy gland [1] is highly prevalent in many regions of the world, resulting in huge and significant economic losses in agribusiness since it is responsible for a decrease in wool production and carcass quality [2]. Mainly small ruminant populations like sheep and goats, and other mammals, such as bovines, pigs, deer, ovines, equines, and even, though rarely, in camels and humans, are the victims of *Corynebacterium pseudotuberculosis* [3-6]. The disease is characterized by the presence of caseous necrosis in external and/or internal lymph nodes [1,7]. Ulcerative lymphangitis, which is confined to the lymph vessels of extremities particularly the hind legs, is a disease caused by this bacterium in the horse [8,9]. The bacterium in some cases of human lymphadenitis, clinical strains are occasionally recovered [10]. The prevalence of CLA in the animals scattered throughout the globe needs effective measures to control the onset of the disease in herds along with the treatment of infected animals. Numerous reports have been published worldwide where mainly small ruminants are the carriers of the *C. pseudotuberculosis*. They include South Africa, Brazil, United States of America, Canada, Australia, New Zealand, United Kingdom and Egypt [11-18]. Histopathological examination of antelope carcasses from a South African game reserve, a part of their routine meat inspection, showed tuberculosis-like lesions. These lesions were characterized by the presence of encapsulated necrogranulomatous inflammation similar to CLA within the pulmonary tissues, in bronchial lymph nodes, liver, kidney and some other organs of the antelopes [11]. Diseases caused by the bacterium *C. pseudotuberculosis* are presented in various clinical forms as sheep and goats, affected with CLA [19]. Among the affected animal population, the increased prevalence and rapid transmission of the disease necessitates certain measures to control disease dissemination and prevent the nearby wildlife. The analysis of the genome sequence will help us better understand the molecular and genetic basis of virulence of this bacterium.

## Classification and Features

*C. pseudotuberculosis* is a facultative intracellular pathogen showing pleomorphic forms like coccoids and filamentous rods, with sizes ranging between 0.5-0.6 µm and 1.0-3.0 µm [2]. Cells are described as Gram-positive, non-encapsulated, non-motile, non-sporulating and possessing fimbriae [12,20]. The bacterium was first isolated in 1888 from bovine farcy by Nocard and was first completely described by Preisz, showing its resemblance to diphtheria bacillus. The organism has been previously named *Bacillus pseudotuberculosis ovis; Bacillus pseudotuberculosi* and*, Corynebacterium ovis* [8,21]. It is a facultative anaerobe. The best growth temperature and pH are 37^o^ C and 7.0-7.2, respectively [17,22]. After initially growing sparsely, strain P54B96 forms organized clumps on the agar surface, demonstrating dry opaque and concentrically ringed colonies. In liquid media it develops a granular deposit with a surface pellicle [8,22,23].

There exist two biotypes of *C. pseudotuberculosis* according to their capability of nitrate reduction. Bacteria capable of performing the reduction of nitrate are classified into biovar *equi* (nitrate reduction positive; mainly isolated from horses and cattle) while the bacteria which can not perform the reduction of nitrate, pertain to biovar o*vis* (nitrate reduction negative; frequently isolated from sheep and goats) [2,24]. Corynebacteria possess an unusual structural organization in their cell envelope, similar to the Gram-negative bacteria [25] and belong to a very heterogeneous CMNR (*Corynebacterium, Mycobacterium, Nocardia* and *Rhodococcus*) group that shares characteristics including an outer lipid layer, mycolic acids in the cell wall along with with its derivatives including phospholipids and lipomannans [4]. Marchand *et al*. (2012) and others reported the presumed mycomembrane, an atypical outer membrane, pore-forming proteins like PorA and PorB, mycoloyltransferases, the so-called fibronectin-binding proteins like cMytA-D and cMytF, several lipoproteins and some unknown putative C-terminal hydrophobic anchored proteins [26]. Analysis of amino acids and amino sugars of cell wall peptidoglycan reveals the presence of *meso*-diaminopimelic acid (*meso*-DAP). Major cell wall sugars are arabinose and galactose [17,27]. In addition, high and low molecular mass glucan, arabinomannan and lipoglycan also make part of the cell wall. Trehalose dimycolate (TDM) and trehalose monomycolate (TMM) are soluble cell envelope lipids [28]. Biochemically, all strains produce acid from glucose, maltose, fructose, sucrose and mannose [21,22]. This bacterium is catalase positive and phospholipase D, beta-hemolysis and oxidase negative [23,29].

**Figure 1:**
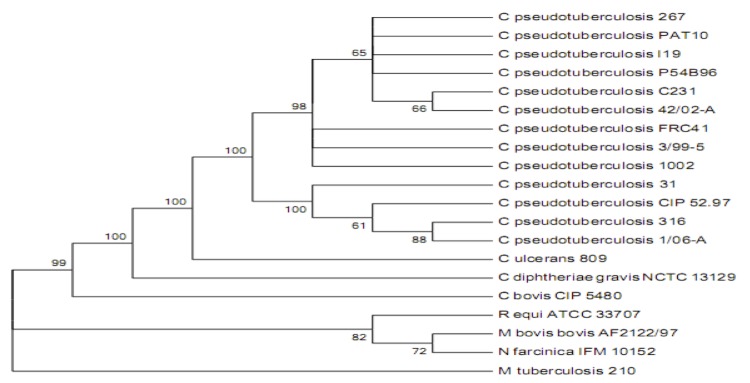
Phylogenetic tree of *C. pseudotuberculosis* strain P54B96 representing its position relative to type strains in *Corynebacteriaceae* along with some other type strains of CMNR group. The tree was inferred from 3,537 aligned characters of the *rpoB* gene sequence using maximum likelihood method and then checked for its agreement with the current classification Table 1. The branch lengths represent the expected number of substitutions per site. Numbers adjacent to the branches are support values from 1,000 bootstrap replicates, indicated when Larger than 60%. Calculations to determine the phylogenetic distances were done by the software MEGA v5 [30].

Figure 1 shows the phylogenetic neighborhood of *C. pseudotuberculosis* strain P54B96 in an *rpoB* gene (β subunit of RNA polymerase) based tree. It has recently been shown that phylogenetic analysis for the identification of *Corynebacterium* as well as other CMNR species based on *rpoB* gene sequences are more accurate than analyses based on 16S rRNA [42,43]. The *rpoB* gene sequences of reference strains from the CMNR group were used to construct the phylogenetic tree.

**Table 1:** Classification and general features of *C. pseudotuberculosis* strain P54B96 according to the MIGS recommendations [31].

**MIGS ID**	**Property**	**Term**	**Evidence code**
	Classification	Domain *Bacteria*	TAS [32]
		Phylum *Actinobacteria*	TAS [33]
		Class *Actinobacteria*	TAS [34]
		Order *Actinomycetales* *Suborder Corynebacterineae*	TAS [34-37]
		Family *Corynebacteriaceae*	TAS [34,35,37,38]
		Genus *Corynebacterium*	TAS [35,38,39]
		Species *Corynebacterium pseudotuberculosis*	TAS [35,40]
		Strain P54B96	TAS [11]
	Gram stain	Positive	TAS [21]
	Cell shape	pleomorphic forms	TAS [21]
	Motility	non-motile	TAS [8]
	Sporulation	non-sporulating	TAS [22]
	Temperature range	mesophilic	TAS [8,22]
	Optimum temperature	37°C	TAS [8,22]
	Salinity	not reported	NAS
MIGS-22	Oxygen requirement	aerobic and facultatively anaerobic	TAS [8,22]
	Carbon source	glucose, fructose, maltose, mannose, and sucrose	TAS [8]
	Energy source	Chemoorganotroph	TAS [8]
MIGS-6	Habitat	Host	TAS [22]
MIGS-15	Biotic relationship	intracellular facultative pathogen	TAS [22]
MIGS-14	Pathogenicity	sheep, goats, horses and cattle, rarely humans	TAS [5,6]
	Biosafety level	2	TAS [22]
	Isolation	liver, lung, mediastinal lymph node lesions of antelope	TAS [11]
MIGS-4	Geographic location	Mpumalanga province, South Africa	TAS [11]
MIGS-5	Sample collection time	2009	TAS [11]
MIGS-4.1 MIGS-4.2	Latitude Longitude	not reported not reported	
MIGS-4.3	Depth	not reported	
MIGS-4.4	Altitude	not reported	

Evidence codes - IDA: Inferred from Direct Assay (first time in publication); TAS: Traceable Author Statement (i. e. a direct report exists in the literature); NAS: Non-traceable Author Statement (i. e. not directly observed for the living, isolated sample, but based on a generally accepted property for the species, or anecdotal evidence). These evidence codes are from the Gene Ontology project [41]. If the evidence code is IDA, then the property was directly observed for a living isolate by one of the authors or an expert mentioned in the acknowledgements.

## Genome sequencing and annotation

### Genome project history

This organism was selected for sequencing on the basis of its phylogenetic position. The genome project is deposited in the Genomes OnLine Database [44] and the complete genome sequence is available in GenBank (CP003385.1). Sequencing, finishing and annotation were performed by the Rede Paraense de Genômica e Proteômica (RPGP), Pará, Brazil. A summary of the project information is shown in Table 2.

**Table 2:** Genome sequencing project information

**MIGS ID**	**Property**	**Term**
MIGS-31	Finishing quality	Finished
MIGS-28	Libraries used	Fragments (mean size 112 bp)
MIGS-29	Sequencing platforms	Semiconductor Ion Torrent PGM
MIGS-31.2	Sequencing coverage	35-fold
MIGS-30	Assemblers	CLC Genome Workbench 4.7.2, Velvet
MIGS-32	Gene calling method	Glimmer v3.02
	INSDC ID	CP003385 (chromosome)
	GenBank Date of Release	April 05, 2012
	GOLD ID	Gc02176
	NCBI project ID	77871
	Database: IMG-GEBA	2512564058
MIGS-13	Source material identifier	BHI broth, P54B96
	Project relevance	Animal Pathogen, Medical

### Growth conditions and DNA isolation

*C. pseudotuberculosis* P54B96 was grown in brain-heart-infusion broth (BHI-HiMedia Laboratories Pvt. Ltda, India) in shake culture at140 rpm and at 37^o^C. Extraction of chromosomal DNA was performed by using 50 mL of 48–72 h culture of *C. pseudotuberculosis*, centrifuged at 4^o^C and 2000× g for 20 min. Re-suspension of cell pellets was done in 1 mL Tris/EDTA/NaCl [10 mM Tris/HCl (pH7.0), 10 mM EDTA (pH8.0), and 300 mM NaCl] for re-centrifugation under the same conditions. The pellets were re-suspended in 1 mL TE/lysozyme [25 mM Tris/HCl (pH8.0), 10 mM EDTA (pH8.0), 10 mM NaCl, and 10 mg lysozyme/mL]. The sample was then incubated at 37^o^C for 30 min and then 30 µL of 30% (w/v) sodium N- lauroyl-sarcosine (Sarcosyl) was added to it, incubated for 20 min at 65^o^C, followed by incubation for 5 min at 4^o^C. Purification of DNA with phenol/chloroform/isoamylalcohol (25:24:1) was followed by precipitation with ethanol. DNA concentration was determined by spectrophotometer, and the DNA was visualized in ethidium bromide-stained 0.7% agarose gel.

### Genome sequencing and assembly

The complete genome sequence of *C. pseudotuberculosis* P54B96 was obtained using the Ion Torrent PGM (Life Technologies) Sequencing Platform. A total, of 562,812 reads were generated, each with a mean size of 112 nts usable sequence (35-fold coverage). Furthermore, a hybrid de novo assembly approach was applied using 376,642 Ion filtered reads (19-fold coverage). This was carried out after quality filtering process during which reads representing an average Phred quality of less than 20, were removed. This strategy allowed closing gaps without bench work time cost [45].

For homopolymer correction, an inherent problem of the Ion Torrent [46], CLCBio Genome Workbench 4.7.2 was used. Having detected a high number of frameshifts, manual curation was required prior to analysis to prevent false-positive identification of pseudogenes. The genome of P54B96 strain consists of 2,337,657 bp circular chromosome and the average G+C content of the chromosome is 52.2%. The genome was predicted to contain 2,084 coding sequences (CDS), four rRNA operons, 49 tRNA and 62 pseudogenes.

### Genome annotation

For automatic annotation, different programs were used. These include; Glimmer: gene predictor [47], RNAmmer: rRNA predictor [48]; tRNA-scan-SE: tRNA predictor [49]; and Tandem Repeat Finder: repetitive DNA predictor [50]. Functional annotation was performed by similarity analyses, using public databases of National Center for Biotechnology Information (NCBI) non-redundant database, Pfam and InterProScan software [51], which integrates multiple domain and protein family databases. Manual annotation was performed using Artemis [52].

### Metabolic network analysis

The metabolic Pathway/Genome Database (PGDB) was computationally generated using Pathway Tools software version 15.0 [53] and MetaCyc version 15.0 [54], based on annotated EC numbers and a customized enzyme name mapping file. There has been no manual curation in the database and it may contain errors, similar to a Tier 3 BioCyc PGDB [55].

## Genome properties

**Figure 2:**
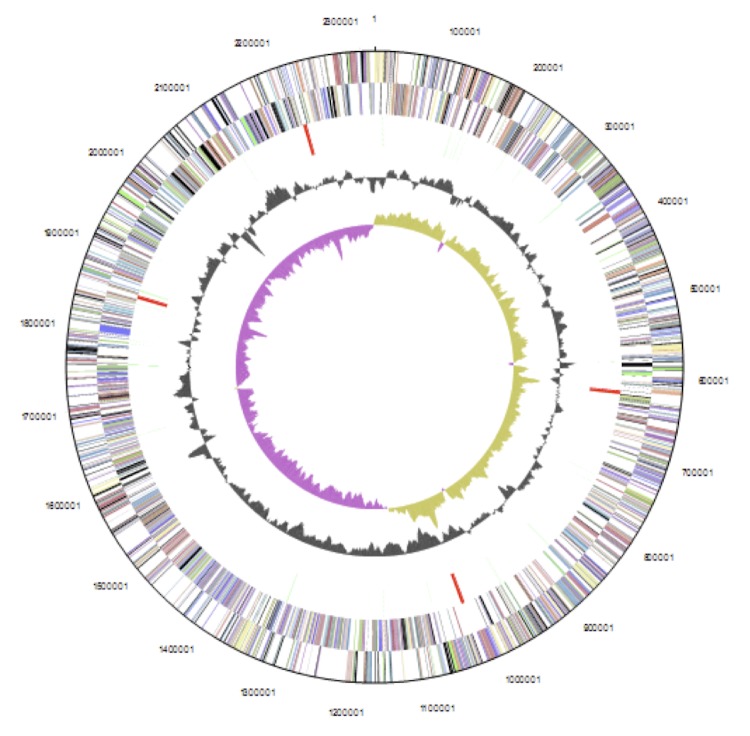
Graphical circular map of the genome. From outside to the center: Genes on forward strand (color by COG categories), Genes on reverse strand (color by COG categories), RNA genes (tRNAs green, rRNAs red, other RNAs black), GC content, GC skew.

**Figure 3:**
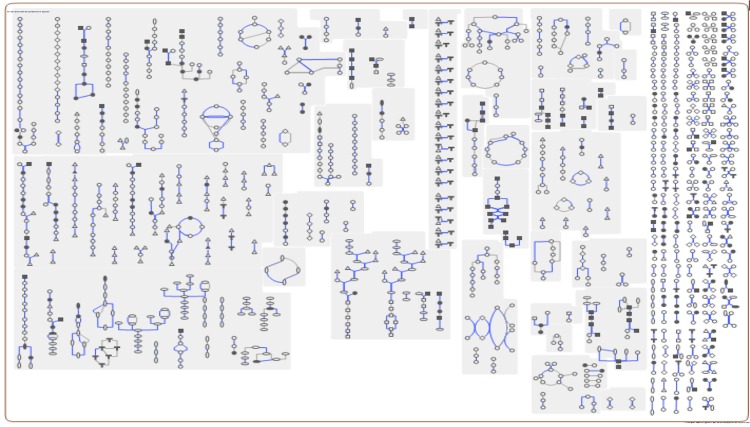
Schematic cellular overview of all pathways of the *C. pseudotuberculosis* P54B96 metabolism. Nodes represent metabolites, with shape indicating class of metabolite. Lines represent reactions.

The genome is 2,337,657 bp long and comprises one main circular chromosome with a 52.19% GC content. A total of 2,207 genes were predicted, among which 2,146 were protein coding genes, and 61 RNAs; 62 pseudogenes were also identified. Of the whole genome, 69.01% comprise genes that were assigned with putative functions, while the remaining genes were annotated as hypothetical proteins. The properties and statistics of the *C. pseudotuberculosis* genome are listed in Table 3. The distributions of genes into COGs functional categories is presented in Figure 2 and Table 4, followed by a cellular overview diagram in Figure 3 and a summary of metabolic network statistics shown in Table 5.

**Table 3:** Genome Statistics

**Attribute**	**Value**	**% of Total**
Genome size (bp)	2,337,657	100.00%
DNA coding region (bp)	2,005,391	85.79%
DNA G+C content (bp)	1,219,912	52.19%
Number of replicons	1	
Extrachromosomal elements	0	
Total genes	2,145	100.00%
RNA genes	61	2.76%
rRNA operons	4	
Protein-coding genes	2,084	97.16%
Pseudo genes	62	2.81%
Genes with function prediction	1,511	68.46%
Genes in paralog clusters	425	19.26%
Genes assigned to COGs	1,552	70.32%
Genes assigned Pfam domains	1,596	72.32%
Genes with signal peptides	651	29.50%
Genes with transmembrane helices	584	26.46%
CRISPR repeats	0	

**Table 4:** Number of genes associated with the general COG functional categories

**Code**	**Value**	**%age**	**Description**
J	140	6.72	Translation, ribosomal structure and biogenesis
A	1	0.1	RNA processing and modification
K	121	5.8	Transcription
L	88	4.2	Replication, recombination and repair
B	0	0.0	Chromatin structure and dynamics
D	21	1.0	Cell cycle control, cell division, chromosome partitioning
Y	0	0.0	Nuclear structure
V	25	1.2	Defense mechanisms
T	54	2.6	Signal transduction mechanisms
M	87	4.2	Cell wall/membrane biogenesis
N	1	0.1	Cell motility
Z	0	0.0	Cytoskeleton
W	0	0.0	Extracellular structures
U	27	1.3	Intracellular trafficking and secretion
O	77	3.7	Posttranslational modification, protein turnover, chaperones
C	90	4.3	Energy production and conversion
G	113	5.4	Carbohydrate transport and metabolism
E	177	8.5	Amino acid transport and metabolism
F	73	3.5	Nucleotide transport and metabolism
H	102	4.9	Coenzyme transport and metabolism
I	57	2.7	Lipid transport and metabolism
P	122	5.9	Inorganic ion transport and metabolism
Q	26	1.3	Secondary metabolites biosynthesis, transport and catabolism
R	169	8.1	General function prediction only
S	136	6.5	Function unknown
-	655	31.4	Not in COGs

**Table 5:** Metabolic Network Statistics

**Attribute**	**Value**
Total genes	2,145
Enzymes	500
Enzymatic reactions	764
Metabolic pathways	152
Metabolites	622
